# Changes and Determinants of Maternal Health Services Utilization in Ethnic Minority Rural Areas in Central China, 1991–2015: An Ecological Systems Theory Perspective

**DOI:** 10.3390/healthcare11101374

**Published:** 2023-05-10

**Authors:** Changli Zhang, Jun Lu

**Affiliations:** School of Public Health, Fudan University, Shanghai 200032, China

**Keywords:** determinants, maternal health services, service utilization, ethnic minority rural areas, China, ecological systems theory

## Abstract

Background: Universal maternal health coverage is a proven, effective strategy for maternal survival. This study aimed to describe the changes and determinants of maternal health service use between 1991 and 2015 in central China. Methods: The study was conducted in Enshi Prefecture. Women were eligible for inclusion if they were rural women who lived in villages, had live births during 1991–2015, could recall their maternal care histories, and had no communication problems. This retrospective study included 470 rural women in 9 villages and collected 770 records. The conceptual framework was designed based on the Society Ecosystem Theory. The determinants included micro-factors (individual characteristics), meso-factors (family factors, community factors, healthcare factors), and macro-factors (government-run maternal and child health programs, abbreviated as MCH programs). Multivariate logistic regressions were applied to analyze the determinants of maternal health service utilization. Results: The utilization of maternal healthcare has improved in Enshi. The hospital birth rate was 98.1% in 2009 and mostly 100% in subsequent years. The prenatal examination rate, the postpartum visit rate, and the continuum of maternal health service (CMHS) rate increased to 73.3%, 67.7%, and 53.4%, respectively, in 2009–2015. The utilization of maternal health services was affected by macro-factors, meso-factors, and micro-factors, with macro-factors being the most notable contributors. Conclusions: Despite the remarkable improvements in antenatal care (ANC) use and hospital birth, gaps in postpartum visits remain. Promoting the integrated continuum of maternal and child healthcare in ethnic minority rural areas requires the joint efforts of the government, health and other sectors, communities, families, and individuals.

## 1. Introduction

For decades, improving maternal health has been one of the WHO’s key priorities, linked to efforts to achieve universal maternal health coverage [1]. Ending preventable maternal death (EPMM) remains an important part of the Sustainable Development Goals (SDGs) [2]. Although the maternal mortality ratio (MMR) dropped by 45 percent worldwide [3] between 1990 and 2015, profound disparities in maternal health persist within and across countries and regions, especially in ethnic minority areas. A total of 94% of maternal deaths occur in low- and lower-middle-income countries [4,5], and 191 (6.7%) counties remained above the SDG target in 2015 in remote western China [6]. Overwhelming evidence indicates that most maternal deaths are preventable through the utilization of maternal health services [7,8,9,10,11,12]. In this study, universal maternal health coverage is defined as the universal health coverage of maternal health services to ensure all women have access to high-quality maternity care. Consequently, universal maternal health coverage is regarded as an effective strategy for EPMM and promoting health and well-being.

Since the 1990s, the Chinese government has been committed to improving maternal health and has introduced several public policies and actions with different priorities in each period. In the 1990s, China launched the Law on Maternal and Infant Health Care, the Outline of Women’s Development, and the Outline of Children’s Development, which protect the rights of mothers and children and ensure they receive appropriate health services [13]. China introduced the strategy of training traditional birth attendants (TBAs) in rural areas. Nevertheless, it showed that MMR did not decline quickly and effectively with the implementation of training TBAs in the 1990s. Then, an institution-based childbirth policy for rural areas was selected as the new national Safe Motherhood policy [14]. During 2000–2008, China actively advocated hospital births. In 2000, China initiated the “Reducing Maternal Mortality and Eliminating Neonatal Tetanus” (RMMENT) program to promote facility births [15] in mid-western China. Until 2013, this program covered 2297 counties with a population of 830 million. The National Health and Family Planning Commission of China issued the New Rural Cooperative Medical Scheme (NCMS) in 2003 and the Rural Hospital Delivery Subsidy in 2008. In 2009, the RMMENT program merged into the National Major Public Health Programs as the Hospital Delivery Subsidy to ensure free hospital birth for all rural women in China [16]. After years of efforts, these actions have led to the transition from TBAs to skilled birth attendants (SBA) in rural China. Since 2009, improving health service continuity and regional equality [17] has become the top national agenda. Thus, the Basic Public Health Service (BPHS) project was launched nationwide in 2009 and focused on providing an essential public health services package that included the establishment of a maternal health record, five antenatal visits [18], and one postpartum visit [19] free of charge at primary health centers [20].

China has made remarkable progress in maternal health [21] and has almost eliminated urban–rural disparities. The rural and urban hospital birth rate was almost the same (99.9% vs. 99.5%) in 2015 [22], and the hospital birth rate in the western region increased from 44.8% in 1996 to 99.7% in 2018 [22]. The MMR declined from 88.8 per 100,000 live births in 1990 [23] to 21.8 per 100,000 live births in 2015 [6] at the national level, achieving MDG 5 on target. According to the 2019 National Health Statistic Yearbook [22], the urban-to-rural ratio of MMR dropped from 1:2.2 in 1990 to 1:1.02 in 2015. Despite the nationwide progress, persistent regional disparities exist between the developed eastern areas and the developing mid-western ethnic minority areas [24,25]. In 2018, MMR in the central and western regions was 1.8 and 2.3 times higher, respectively, than that in the eastern regions [23]. However, Enshi Prefecture in central China has made impressive achievements in improving maternal health and has narrowed the gap with eastern China. Enshi Prefecture is a developing minority mountainous district adjacent to Chongqing and Guizhou Province in western China, which have a similar social, economic, and cultural environment. The hospital birth rate increased from 27.2% in 1999 to 99.8% in 2014. The MMR in 1999 (163.6/100,000) was the highest in Hubei Province; it decreased to 7.1/100,000 in 2014 [26], lower than the national level in 2015 and the eastern region level of 25.2/100,000 in 2018 [23]. The experience of this remote ethnic minority area has significant policy implications for China’s efforts to reduce county-level disparities in maternal health in western regions.

It is well known that the Chinese government has different priorities for maternal health interventions at different times. Continuity of care for maternal health services (CMHS) includes antenatal care (ANC), skilled care during childbirth, and postnatal care (PNC) from pregnancy to 42 days after delivery [27]. Adequate CMHS are critical for reducing maternal mortality [28]. Although there are many cross-sectional studies on ANC [29], hospital deliveries [24], or PNC [30], few articles explore the utilization of CMHS [31] and analyze the change in characteristics in different periods. Inadequate utilization of maternal health services remains severe in remote ethnic minority areas [32]. According to the ecological systems theory, the environment that affects health behavior can be divided into the microsystem, the mesosystem, and the macrosystem [33,34]. Although the literature has focused more on the micro- and meso-factors of maternal health service use, such as sociodemographic characteristics, education, household wealth, and the health workforce [35], few studies have conducted robustness analyses on macro-factors such as policies [36,37,38]. More population-based longitudinal studies are needed.

This study aimed to describe the changes and determinants of maternal health service use between 1991 and 2015 in Enshi Prefecture.

## 2. Materials and Methods

### 2.1. Study Settings

We chose to focus on Enshi Tujia and Miao Autonomous Prefecture, a region where minority nationality, poverty, and geographical and cultural access resulted in vast challenges in improving maternal health coverage in the 1990s. Autonomous prefectures, which are prefecture-level ethnic autonomous administrative divisions established in areas where ethnic minorities live, enjoy a high degree of self-management authority in China. Enshi Prefecture is the only ethnic minority autonomous prefecture in Hubei Province [39], located in the southwest mountainous region. It has two county-level cities and six counties, which are poverty-stricken. Enshi Prefecture is a multi-ethnic area with a total population of nearly 4 million, with Han Chinese accounting for 46% and ethnic minorities accounting for 54%. The Tujia and Miao account for the highest percentage of ethnic minorities.

### 2.2. Study Design and Sample

A population-based retrospective study was applied to collect information on women’s maternal healthcare histories. Women were eligible for inclusion if they were rural women who lived in villages, had live births during 1991–2015, could recall their maternal care histories, and had no communication problems. The exclusion criteria were urban women and state employees working in rural areas.

We used a three-stage stratified sampling method to collect the data. First, we chose Lichuan by purposive sampling. The key informant in the Prefecture Maternal and Child Health Center believed that Lichuan, with the largest population, was the most representative. Second, we randomly chose three towns based on their economic level. Third, we randomly selected nine villages among the three townships based on their distance from the township, and about fifty women were surveyed in each village. The village doctors selected the respondents. The respondents completed the questionnaire at the village clinic, village committee, or their home. We investigated 476 women, out of whom 470 questionnaires were valid. The response rate was 98.7%. Finally, we collected 770 pieces of maternal healthcare history information from 470 women who gave birth during 1991–2015.

This study was conducted in August 2015 using a structured questionnaire. Basic information on sociodemographic characteristics and the utilization of maternal health services was collected. Trained undergraduate students and staff from local medical facilities conducted the questionnaire survey through face-to-face visits. After participants were introduced to the informed consent form in the field and signed it, the investigators interviewed them individually to collect data.

### 2.3. Variables

#### 2.3.1. Dependent Variables

The dependent variables included four variables indicating maternal health service utilization. According to the Guideline for Maternal Health Care Service (GMHCC) of China, the utilization of the continuum of maternal health service (CMHS) refers to women who attended at least five prenatal examinations, had hospital births, and received at least one postnatal visit from pregnancy to 42 days after delivery [40]. Thus, the dependent variables were prenatal examination rate (≥5 visits), hospital birth rate, postpartum visit rate (≥1 visit), and CMHS rate.

#### 2.3.2. Independent Variables

**Figure 1:**
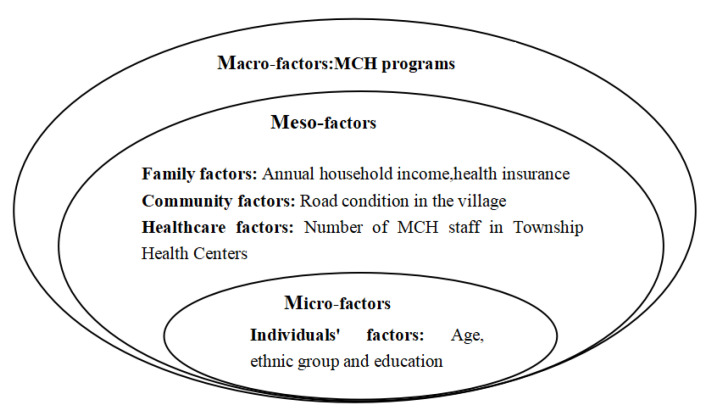
Conceptual framework of maternal health service utilization determinants.

Based on the Society Ecosystem Theory [41], this study constructed a conceptual framework (Figure 1) of maternal health service utilization determinants. The ecological environment is conceived as a set of nested structures; the outer-layer systems affect the inner-layer systems. The term microsystem refers to the individual systems, including the physiological and psychological subsystems. The term mesosystem refers to small-scale groups associated with individuals, such as communities, families, and other social groups. The term macrosystem refers to social systems larger than small-scale groups, including culture, policy, etc. Therefore, we defined three dimensions of independent variables: micro-factors (individual characteristics), meso-factors (family factors, community factors, healthcare factors), and macro-factors (government-run maternal and child health programs, abbreviated as MCH programs).

### 2.4. Data Analysis

First, the database was checked for outliers and missing data and cleaned. Then, we performed a descriptive analysis to demonstrate the characteristics of the study population and changes in maternal service utilization rate before 2000, during 2000–2008, and after 2009. The Safe Motherhood strategies for the three periods were training traditional birth attendants, institutional deliveries, and promoting universal coverage of maternal health care, respectively. Finally, multivariate logistic regression was applied to analyze the associations between maternal health care utilization and determinants; odds ratios (OR) and 95% confidence intervals (CI) were calculated, and the enter method was selected, with a value of *p* < 0.05 considered statistically significant. We performed statistical analyses with SPSS version 24.0 (IBM, New York City, NY, USA).

### 2.5. Ethical Approval

The research was approved by the Medical Research Ethics Board of the School of Public Health, Fudan University (IRB00002408&FWA00002399), and the accreditation number was IRB#2015-07-0557. The approval date was 20 July 2015. All participants were informed and signed a written informed consent.

## 3. Results

### 3.1. Characteristics of the Study Population

The respondents included 227 ethnic minorities and 243 Han women. Most respondents had low levels of education, and only 24.5% of women had a high school diploma or above.

Table 1 shows the changes and descriptions of the characteristics of mothers during different periods. The percentage of women below 25, with education below high school, and annual household income below CNY 10,000 declined between 1991 and 2015. The health insurance coverage, the road conditions in the village, and the number of MCH professionals in township health centers gradually improved.

### 3.2. Changes in Maternal Services Utilization in Enshi, 1991–2015

**Figure 2:**
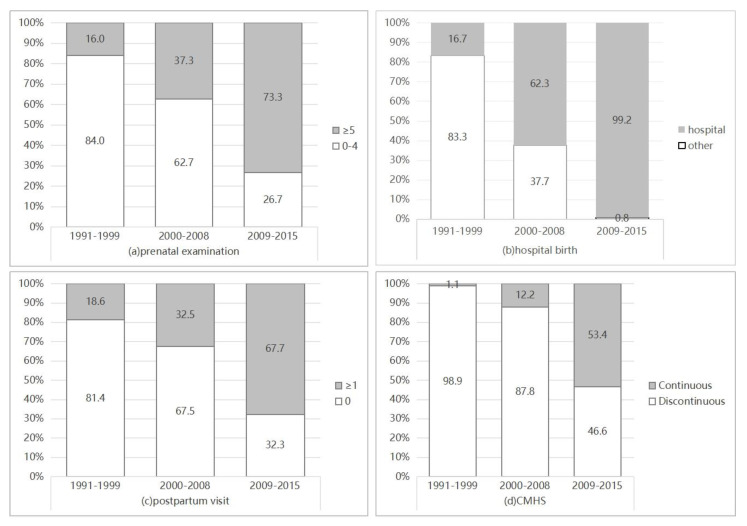
Changes in maternal services utilization rate (%), 1991–2015. (**a**) Changes in prenatal examination rate (≥5 visits); (**b**) changes in hospital birth rate; (**c**) changes in postpartum visit rate (≥1 visit); (**d**) changes in CMHS rate.

Figure 2 shows the changes in the percentage of maternal healthcare utilization during different periods. The prenatal examination rate increased from 16.0% in 1991–1999 to 37% in 2000–2008, then increased to 73.3% in 2009–2015. The hospital birth rate rose dramatically and gained almost universal coverage. The hospital birth rate increased to 98.1% in 2009 and was 100% for all years between 2010 and 2015 except for 2011 (97.7%). The postpartum visit rate has changed from 18.6% to 67.7% in the past 25 years. Only 1.1% of rural women utilized the continuum of maternal health services in 1991–1999, but in 2009–2015, the CMHS rate increased to 53.4%.

In the 1990s, the utilization rate of maternal healthcare in Enshi Prefecture was low. During the duration of the RMMENT program implemented in 2000–2008, despite the hospital delivery rate increasing rapidly, the prenatal examination and postpartum visit rates remained low, so the CMHS rate was still not high. Since the BPHS program launched in 2009, both prenatal health care and postpartum care have improved, and the CMHS rate has increased rapidly.

### 3.3. Determinants of Maternal Healthcare Utilization

Table 2 presents regression estimates on factors affecting maternal healthcare utilization.

#### 3.3.1. Factors Associated with Prenatal Examinations

It was found that higher annual household income (OR = 1.743, 95% CI: 1.201~2.531; OR = 1.784, 95% CI: 1.012~3.143), good road conditions in the village (OR = 1.562, 95% CI: 1.023~2.385), higher numbers of MCH staff in the township health center (OR = 1.198,95% CI: 1.031~1.390), and the BPHS project (OR = 4.510,95% CI: 1.743~11.666) had a positive association with the prenatal examination rate. Compared with the 1990s, the odds ratio of the prenatal examination rate was 4.5 times higher during the implementation of the BPHS project.

#### 3.3.2. Factors Associated with Hospital Births

Hospital delivery was significantly associated with higher education levels (OR = 3.234, 95% CI: 1.420~7.368), health insurance (OR = 5.780, 95% CI: 2.773~12.048), good road conditions in the village (OR = 2.421, 95% CI: 1.406~4.171), higher numbers of MCH staff in the township health center (OR = 1.659, 95% CI: 1.269~2.169), the RMMENT program (OR = 2.718, 95% CI: 1.200~6.155), and the BPHS project (OR = 21.891, 95% CI: 3.655~131.117).

#### 3.3.3. Factors Associated with Postpartum Visits

The utilization of postpartum visits was significantly associated only with the number of MCH staff in the township health center (OR = 1.305, 95% CI: 1.128~1.510).

#### 3.3.4. Factors Associated with the Continuum of Maternal Health Service

It was found that higher education levels (OR = 1.928, 95% CI: 1.248~2.977), higher annual household income (OR = 1.657, 95% CI:1.078~2.545; OR = 1.958, 95% CI: 1.115~3.441), good road conditions in the village (OR = 2.193, 95% CI: 1.231~3.907), higher numbers of MCH staff in the township health center (OR = 1.393, 95% CI: 1.191~1.630), and the BPHS project (OR = 8.949, 95% CI: 1.087~73.648) had a positive association with the CMHS rate.

## 4. Discussion

### 4.1. Toward Universal Maternal Health Coverage: Progress and Gap

Enshi Prefecture achieved notable progress in universal access to SBA and ANC earlier than rural western China [42]. Ensure Skilled Attendance at Delivery has become an international consensus on Safe Motherhood. The hospital birth rate in 2008 increased to 91.5%, 30% higher than the most remote rural region in China [43], better than in 2016 in Ethiopia [28] and 2013 in India [44]. The hospital birth rate rapidly increased by 200.0% during 2000–2015, with an annualized rate of 13.5% during 2000–2008 and 0.3% during 2009–2015. Koblinsky et al. [45] described four models of care: home deliveries by community members (Model 1), home deliveries by a professional attendant (doctor or midwife) (Model 2), delivery by professional attendants in a basic Essential Obstetric Care (EOC) facility (Model 3), and delivery by professionals in a comprehensive EOC facility (Model 4). When reviewing the last 30 years (1987–2017), the promoted model of care within low-resource settings has progressed from Models 1 and 2 in the first decade to Model 4 in the third decade [46]. Each country should choose the appropriate strategy in different social and cultural contexts. Though countries such as China, Malaysia, and Sri Lanka have selected the institutional delivery model and achieved great progress, not all countries are ready to adopt arguably the most advanced Model 4, and its affordability by many developing countries is doubtful [45].

According to the WHO, the prenatal examination rate (≥4 visits) in Enshi was higher than that in South Asia (42%) [47], Indonesia (78%) [48], and Liberia (42.5%) [49]. Then, it increased to 97.4% in 2015, higher than 64% globally [36]. According to GMHCC, the prenatal examination rate (≥5 visits) increased to 87.2% in 2015, 5.7 times higher than in 1999 and better than in eastern rural China in 2013 (79.8%) [50]. However, there were still over 10% who failed to attend five or more antenatal visits. Furthermore, most studies [51], including the present one, did not define the timing of antenatal care.

Postnatal care is currently the weakest gap in the maternal health care continuum, making the CMHS rate difficult to improve. In low- and middle-income countries (LMICs), antenatal care use is generally high, while postnatal care use is low [36]. The postpartum visit rate (≥1 visit) increased from 24.0% in 1999 to 82.5% in 2015, higher than in 2014 in western Sichuan (28.4%) [32]. However, only 10% of women received ≥3 postpartum visits within 42 days of delivery in 2015, lower than in 2013 in rural China (25.3%) [30]. According to GMHCC, the CMHS rate increased from 3.0% in 1999 to 72.5% in 2015. However, according to the WHO, the CMHS rate was still low (10%) in 2015. Despite the impressive progress in universal access to institutional delivery and prenatal care, there are still many challenges in postnatal care, such as the lack of skilled human resources and ignorance of PNC benefits [52]. The results of the regression analysis show that the impact of micro- and macro-factors on the postpartum visit rate is not statistically significant, which suggests that we need to provide health education to rural women and improve the efficiency and effectiveness of MCH policy implementation. In the future, China should focus on postpartum care and its role in linking maternal and child health care to improve continuity of care.

### 4.2. The Determinants of Maternal Healthcare Utilization

This study confirmed that the utilization of maternal health services was affected by macro-factors, meso-factors, and micro-factors, with macro-factors being the most notable contributors. We found that the MCH programs were associated with the use of ANC, hospital deliveries, and CMHS. The RMMENT program, the Chinese version of the Safe Motherhood Program, reflected the government’s strong political will and contributed to institutional births, thus narrowing the maternal health gap between urban and rural areas. Consistent with other studies, this study showed that the government-funded BPHS project contributed to prenatal examination rates [38], CMHS rates [31], and the hospital birth rate. We identified that the BPHS project was not significantly associated with postpartum visits, which is not in line with the findings of another study [38]. Previous studies found that the RMMENT program and the BPHS project reduced maternal mortality [6,8,13,53,54]. The effectiveness of these MCH programs confirmed the importance of government commitment and political will in improving maternal health [55], which is consistent with the experience of countries such as Malaysia and Sri Lanka [45]. To meet the SDGs, China also needs to adjust the main tasks of its MCH programs, including targeting the promotion of postnatal care use and improving the quality of maternal health services.

The most important meso-factor is MCH staff in township health centers. Our findings showed that the increasing number of MCH personnel in township hospitals contributed to ANC, hospital deliveries, PNC, and CMHS and was the only determinant for postpartum visits. Commonly known as the engines of health systems in much of the developing world, community health workers connect the patient to the health system [56]. Despite the number of health personnel increasing in primary health facilities [57], the workload of MCH staff has increased substantially after China’s 2009 healthcare reform. Thus, the MCH workforce shortage [58] and inadequate professional skills [59] remain challenges to the growing demand for high-quality maternal health services. Therefore, it is vital to allocate sufficient MCH personnel at the grassroots level [50] to strengthen postnatal care, particularly female staff and village MCH workers. In the future, China should continue to increase investment to attract staff to primary health facilities and improve their professional capacity through various forms of training.

Another important meso-factor are the road conditions in the village. The regression analysis indicated that road conditions in the village affect the utilization of ANC, hospital deliveries, and CMHS, which suggests the significant role of factors outside the health system in promoting maternal health. Poor transport infrastructure, such as the lack of transport and roads, is one of the main barriers to ANC and hospital birth in ethnic rural areas [24,32], and systematic improvements in road conditions have contributed to the improvements in maternal health [6]. Poor road conditions not only reduce the geographic accessibility of maternal health services or lead to the incidence of transportation delays in emergency referrals but can also affect the behavior of women and village doctors. Women may reduce the frequency of antenatal visits or be reluctant to give birth in the hospital due to concerns about the adverse effects of bumpy travel on the fetus. Village doctors may reduce the frequency of postpartum visits due to inconvenient transportation. Previous studies found that the short-term strategy of providing transportation subsidies [24,58] has been practiced successfully in China and other LMICs. The long-term strategy is to increase government investment and improve transport infrastructure, thus promoting the geographical equity of health services and reducing referral delays [13].

The contribution of micro-factors is weaker than that of other determinants. Different from previous studies in ethnic minority areas in western China [37], this study found that there was no difference in the utilization of prenatal health care services between ethnic minorities and Han women. Health insurance had improved women’s affordable access to hospital births with the RMMENT program and the Rural Hospital Delivery Subsidy [42] before 2009. As China achieved universal health insurance coverage, the inequity of hospital births for rural women diminished. Since 2009, prenatal care and postnatal visits, which are components of essential public health services, have been provided free of charge by the central government, so health insurance has no impact on prenatal care, postnatal visits, or continuity of care. Household economic income had a positive correlation with maternal health service utilization. Among the individual factors, women’s education was positively associated with institutional delivery and continuity of healthcare services, indicating the value of health education.

### 4.3. Limitations

As we know, more studies have focused on changes in maternal health services in ethnic majority areas in western China rather than central China. However, our study has some limitations. First, the sampling size is small. We could not find more respondents in the limited survey time because many mothers worked in the city away from home. Second, the data were self-reported by rural women and may have recall bias. Nevertheless, the recall bias is assumed to be small because pregnancy and childbearing are events that women can remember for years [60]. Third, we did not consider the timing and quality of ANC in this study. To reduce recall bias, we only selected variables that women could accurately remember, such as the number of prenatal visits. Finally, the postpartum visit rates may be underestimated. In the case of institutional deliveries, the mother might receive her first postpartum visit within 48 h of delivery in the hospital, which is easy to forget, resulting in the postpartum visit rate being lower in this study than it actually is. These issues deserve our attention in future studies.

## 5. Conclusions

We found a significant increasing trend in coverage of maternal health services in Enshi Prefecture since 2000 and a gradual reduction in inequality between urban and ethnic minority rural areas during 2000–2015. Despite the remarkable improvements in ANC use and hospital births, gaps in postpartum visits remain. From a life-cycle perspective, we should pay attention to the linkage of prenatal, intrapartum, and postnatal care, especially postnatal visits, to promote the integrated continuum of maternal and child healthcare. MCH programs, MCH personnel in township health centers, transportation, household economic income, and women’s education are the main determinants of maternal health service use found in this study, with MCH programs being the most critical contributors. As a complex social system, the utilization of maternal health care services in remote ethnic minority areas requires the joint efforts of the government, health and other sectors, communities, families, and individuals. These findings have significant implications for policymakers and health service providers. Furthermore, this study sets an example for other low- and middle-income countries to improve maternal health.

## Figures and Tables

**Table 1:** Demographics of women interviewed.

Variables	1991–1999 (n = 94)	2000–2008 (n = 306)	2009–2015 (n = 373)	*p*
	n (%)/$\overset{-}{x}$ ± s	n (%)/$\overset{-}{x}$ ± s	n (%)/$\overset{-}{x}$ ± s	
Micro-factors: Individuals’ characteristics				
Age (years)				
≤24	65 (69.1)	172 (56.2)	143 (38.3)	
25–29	24 (25.5)	81 (26.5)	125 (33.5)	
≥30	5 (5.3)	53 (17.3)	105 (28.2)	<0.001
Ethnic group				
Ethnic minority	54 (57.4)	140 (45.8)	181 (48.5)	
Han	40 (42.6)	166 (54.2)	192 (51.5)	0.140
Education				
Below high school	77 (81.9)	261 (85.3)	272 (72.9)	
High school or above	17 (18.1)	45 (14.7)	101 (27.1)	<0.001
Meso-factors: Family factors				
Annual household income				
≤CNY 10,000	79 (84.0)	178 (58.2)	102 (27.3)	
CNY 10,000–50,000	14 (14.9)	112 (36.6)	182 (48.8)	
>CNY 50,000	1 (1.1)	16 (5.2)	89 (23.9)	<0.001
Health insurance				
No	90 (95.7)	225 (73.5)	27 (7.2)	
Yes	4 (4.3)	81 (26.5)	346 (92.8)	<0.001
Meso-factors: Community factors				
Road conditions in the village				
Poor	90 (95.7)	225 (73.5)	27 (7.2)	
Good	4 (4.3)	81 (26.5)	346 (92.8)	<0.001
Meso-factors: Healthcare factors				
Number of MCH staff in township health center	1.76 ± 1.013	3.48 ± 1.044	5.87 ± 1.411	<0.001

Abbreviations: Han: Han Chinese is the main nationality of China. Han here refers to the Han Chinese women surveyed. MCH: maternal and child health.

**Table 2:** Logistic regression model of maternal healthcare utilization.

Variables	Prenatal Examinations (≥5 Visits)	Hospital Births	Postpartum Visits (≥1 Visits)	CMHS
	OR (95% CI)	OR (95% CI)	OR (95% CI)	OR (95% CI)
Micro-factors: Individuals’ characteristics				
Age (years)				
≤24	Reference	Reference	Reference	Reference
25–29	1.334 (0.852, 2.087)	1.909 (0.912, 3.997)	1.002 (0.647, 1.552)	1.216 (0.751, 1.969)
≥30	1.057 (0.656, 1.704)	0.621 (0.279, 1.383)	1.050 (0.657, 1.677)	1.02 6(0.618, 1.701)
Ethnic group				
Ethnic minority	Reference	Reference	Reference	Reference
Han	1.010 (0.721, 1.414)	1.087 (0.647, 1.827)	0.966 (0.695, 1.341)	1.084 (0.746, 1.573)
Education				
Below high school	Reference	Reference	Reference	Reference
High school or above	1.400 (0.911, 2.153)	3.234 (1.420, 7.368) **	1.499 (0.993, 2.265)	1.928 (1.248,2.977) **
Meso-factors: Family factors				
Annual household income				
≤CNY 10,000	Reference	Reference	Reference	Reference
CNY 10,000–50,000	1.743 (1.201, 2.531) **	1.305 (0.740, 2.302)	1.263 (0.873, 1.829)	1.657 (1.078, 2.545) *
>CNY 50,000	1.784 (1.012, 3.143) *	2.426 (0.490, 12.026)	1.442 (0.831, 2.500)	1.958 (1.115, 3.441) *
Health insurance				
No	Reference	Reference	Reference	Reference
Yes	1.092 (0.704, 1.694)	5.780 (2.773, 12.048) ***	1.197 (0.771, 1.857)	1.377 (0.821, 2.309)
Meso-factors: Community factors				
Road conditions in the village				
Poor	Reference	Reference	Reference	Reference
Good	1.562 (1.023, 2.385) *	2.421 (1.406, 4.171) **	1.073 (0.702, 1.640)	2.193 (1.231, 3.907) **
Meso-factors: Healthcare factors				
Number of MCH staff in township health center	1.198 (1.031, 1.390) *	1.659 (1.269, 2.169) ***	1.305 (1.128, 1.510) ***	1.393 (1.191, 1.630) ***
Macro-factors: MCH programs				
Before RMMENT (before 2000)	Reference	Reference	Reference	Reference
RMMENT (2000–2008)	1.933 (0.891, 4.195)	2.718 (1.200, 6.155) *	0.864 (0.429, 1.741)	4.520 (0.588, 34.752)
BPHS (2009–2015)	4.510 (1.743, 11.666) **	21.891 (3.655, 131.117) **	1.516 (0.627, 3.664)	8.949 (1.087, 73.648) *

* *p* < 0.05,** *p* < 0.01,*** *p* < 0.001. Abbreviations:Han: Han Chinese is the main nationality of China. Han here refers to the Han Chinese women surveyed. MCH: maternal and child health. Before RMMENT (before 2000): in the period before the implementation of the ‘Reducing Maternal Mortality and Eliminating Neonatal Tetanus’ (RMMENT) program, China mainly implemented the strategy of training traditional birth attendants in rural areas. RMMENT (2000–2008): the period when China implemented the RMMENT program and promoted the strategy of institutional delivery in rural areas. BPHS (2009–2015): the period when the Basic Public Health Service (BPHS) project was launched nationwide.

## Data Availability

The authors will supply the relevant data in response to reasonable requests.
